# Human pressures on two estuaries of the Iberian Peninsula are reflected in food web structure

**DOI:** 10.1038/s41598-019-47793-2

**Published:** 2019-08-08

**Authors:** I. Donázar-Aramendía, J. E. Sánchez-Moyano, I. García-Asencio, J. M. Miró, C. Megina, J. C. García-Gómez

**Affiliations:** 1 Laboratorio Biología Marina, Seville Aquarium R + D + I Biological Research Area., Dpto. Zoología, Facultad de Biología, Universidad de Sevilla, Avd. Reina Mercedes 6, 41012 Sevilla, Spain; 2 Dpto. Zoología, Facultad de Biología, Universidad de Sevilla, Avd. Reina Mercedes 6, 41012 Sevilla, Spain; 3 Biodiversidad y Ecología Acuática, Seville Aquarium R + D + I Biological Research Area, Facultad de Biología, Universidad de Sevilla, Avd. Reina Mercedes 6, 41012 Sevilla, Spain

**Keywords:** Ecosystem ecology, Stable isotope analysis, Ecological networks

## Abstract

As a result of the increased urban and agricultural development in coastal environments, estuaries are among the most modified and threatened aquatic ecosystems. This study used stable isotopes to examine the effects of human impacts by contrasting the food web structures of two Iberian estuaries exposed to different degrees of human pressure. More complex feeding pathways were found in the more altered estuary (Guadalquivir). Greater spread among species along the carbon axis suggests that the primary consumers exploit organic matter with various origins, whereas different nitrogen signals of the secondary consumers suggest that they feed on different suites of prey. In contrast, the similar isotopic signals of secondary consumers in the relatively little influenced estuary (Guadiana) suggests similarity in diet composition and feeding on the same organic matter sources. Understanding trophic interactions in estuaries is vital for defining proper management and conservation, and the preliminary data provided here are one step in this direction.

## Introduction

Estuaries are some of the most biologically productive ecosystems in the world^1–3^. They play an important role in the continental shelf environment, acting as nursery habitats and providing other habitats with invaluable ecosystem services^4–6^. However, with more than 60% of Earth’s population living in coastal areas, estuarine ecosystems have been extensively altered by human activities^7^. Rapid urban and agricultural development is the major factor contributing to wetland loss and the deterioration of water quality in these coastal areas^8,9^. Nutrient load inputs to estuaries are directly related to intensive agriculture and large populations^10^ and have the potential to alter nutrient dynamics, in turn modifying the functioning and structure of estuarine ecosystems^11^.

Elevated loads of nutrient input of anthropogenic origin into aquatic ecosystems may affect different ecological processes such as basal resource production, nutrient dynamics and energy transfer^12^. These impacts can alter a system’s trophic structure^12,13^ defined as the distribution of organisms in terms of biomass among producers and consumers^12^. For example, nutrient loading of ecosystems may shift primary production to a single basal source, which is exploited by fewer intermediate consumers, thereby converting a structured and compartmented ecosystem into one with a less stable food web^14–16^. In contrast, these impacts can favour autotrophs and increase the nutritional quality of basal resources^12^. Analysing community trophic structure is one way to assess the nature and magnitude of human impacts^17^. Additionally, trophic niches, which describe the overall trophic role of species within an ecosystem, including all the trophic interactions, and it is often realised as the dietary resource base of consumers^12,18,19^, respond quickly to modification of basal resources and biotic interactions^12,20,21^. These niches therefore provide insights into the functional effects of nutrient loading in aquatic ecosystems.

Stable isotope analysis (SIA) is one of the primary tools used to examine the structure and dynamics of food webs^22^ and may represent a unifying methodology with which to compare anthropogenic pressures among different coastal ecosystems^23^. SIA provide time- and space-integrated information on the trophic interactions of species^18^ in disturbed, undisturbed or restored ecosystems^24^. An example of SIA applicability is the assessment of the effects of invasive species on the trophic structure of native communities. It can be useful to analyse both direct predatory behaviour and indirect impacts on local food webs and to predict potential spread by comparing trophic niche metrics with those of the native species^23,25^. Moreover, stable isotopes is used to track the source of nutrients in a food web, to characterize the trophic niche of species (isotopic niche^18,21^). In this context, analysis of δ^15^N and δ^13^C stable isotope ratios is frequently used in estuarine systems to assess nutrient pollution, organic matter origin and trophic interactions^3,8,26,27^. δ^13^C is typically used to determine the origin of carbon sources^28^. δ^15^N values allow the study of trophic levels of consumers^28^, and the enriched nitrogen isotopic composition of biota can be an indicator of anthropogenic wastewater^27,29^. However, nutrient contamination studies that include multiple taxa and different trophic levels in the food webs are not common^30^.

Another approach based on SIA used to quantitatively characterize the community trophic niche aspects of food webs includes the “Layman metrics^18^”. As Layman *et al*.^18^ proposed, these metrics provide an integrated estimate of multiple anthropogenic-related impacts on food webs, including parameters such as trophic diversity or food web stability and trophic resilience^22,23,25^. While these metrics have been applied in marine ecosystems, their use in estuarine systems remains limited. As Mitchell *et al*. suggested^31^, more studies are needed to assess the implications of this approach as a monitoring and management tool.

In this study, we used SIA to contrast the food web structure of two Iberian estuaries exposed to different degrees of urban and agricultural perturbations. First, we hypothesized that the higher nitrogen isotope values of focal species would reflect greater anthropogenic pressures within the estuary. Second, we hypothesized that human impacts in the more impacted estuary would result in a more homogeneous basal resource pool. Third, we predicted a more complex food web in the less impacted estuary.

### Study system

**Figure 1:**
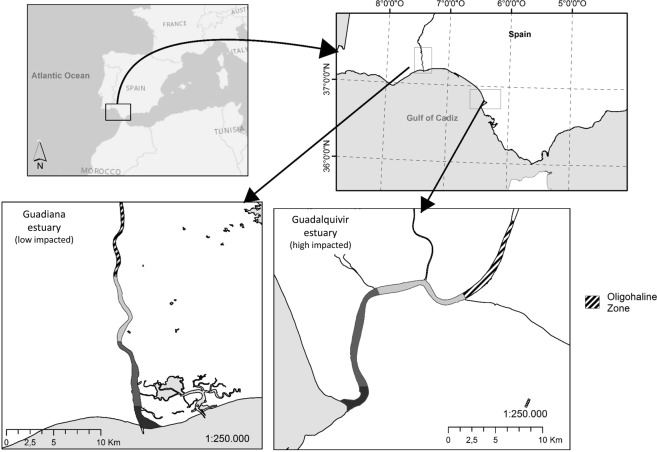
Sampling locations in the oligohaline zone of the Guadiana estuary (left) and Guadalquivir estuary (right). The salinity gradient of the estuaries is provided in grey scale from the euhaline zone (black) to the oligohaline zone (hatched area). The maximum turbidity zone is within the oligohaline zone.

The Guadiana and Guadalquivir Rivers are the largest rivers in the southern Iberian Peninsula. The estuaries of these two rivers are located in the Mediterranean climate region, and both flow to the Gulf of Cadiz on the Atlantic coast (Fig. 1). Both estuaries have hydrological regimes regulated by dams. The flow is low in summer, with episodic freshwater runoff in winter^32,33^. The estuaries are mesotidal and vertically well mixed with a longitudinal salinity gradient. Despite the similarities of these estuaries, they have not been subjected to the same level of disturbances over recent years.

The Guadalquivir estuary is an example of a highly impacted estuarine environment. It crosses extensive rural areas and has been exposed to increasing human activity^34^. Such activity includes desiccation of tidal marshes, isolation of the estuary course from the original tidal marshes, a reduction in freshwater inputs, and eutrophication from urban and agricultural wastes due to continual dredging work^35,36^. All these impacts have caused the Guadalquivir estuary to be characterized by high turbidity levels and increased nutrient loadings^37,38^.

In contrast, the Guadiana estuary is characterized by relatively lower anthropogenic pressures^39,40^. Although it is also influenced by agriculture, agroindustrial activities, and dams^41,42^, this estuary has been catalogued as one of the least polluted European estuaries^42,43^. Furthermore, it is considered one of the best preserved and most vulnerable estuaries of the Iberian Peninsula^41^. Comparative studies of both rivers have found concentrations of N one order of magnitude higher in the Guadalquivir estuary than in the Guadiana estuary associated with the influence of agricultural runoff in the waters^44^, as well as, twice as high pollution in modern sediments of the Guadalquivir estuary^45^. Although there are differences in human pressures between the estuaries, their biological communities contain a very similar set of species^46^. For this reason, the Guadiana estuary has been used as a reference area in other biological studies^39^. To understand human impacts on each estuary, analysis carried out in summer 2017 showed that in the Guadalquivir estuary, ammonia ranged from 0.11 to 0.13 mg/L (0.12 ± 0.012 mg/L mean ± SD), and nitrate ranged from 1.33 to 3.73 mg/L (2.68 ± 1.23 mg/L mean ± SD), while in the Guadiana estuary, the levels were under the detection limits (0.05 mg/L for ammonia and 0.15 mg/L for nitrate). Turbidity in the Guadalquivir estuary is significantly higher than that in the Guadiana estuary (316 ± 94 NTU (Nephelometric Turbidity Unit) in the Guadalquivir estuary, while turbidity in the Guadiana estuary was 80 ± 31.21 NTU).

## Results and Discussion

**Figure 2:**
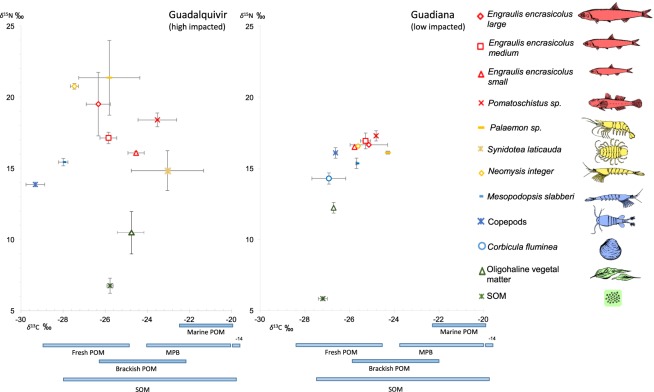
δ^13^C and δ^15^N (mean ± standard deviation) of the primary producers, invertebrates and fishes collected in the Guadalquivir (left) and Guadiana (right) estuaries in summer 2017. Horizontal bars below the x-axis represent the δ^13^C ranges of primary producers extracted from the literature (Supplementary Table S1). POM: particulate organic matter, SOM: sedimentary organic matter, MPB: microphytobenthos (upper limit of MPB range, −14‰, is out of the axis limit). Figures of the different organisms are provided for a better understanding of the species. Colours indicated trophic position: Secondary consumers: red. Primary and/or secondary consumers: yellow. Primary consumers: blue. Producers: green.

**Figure 3:**
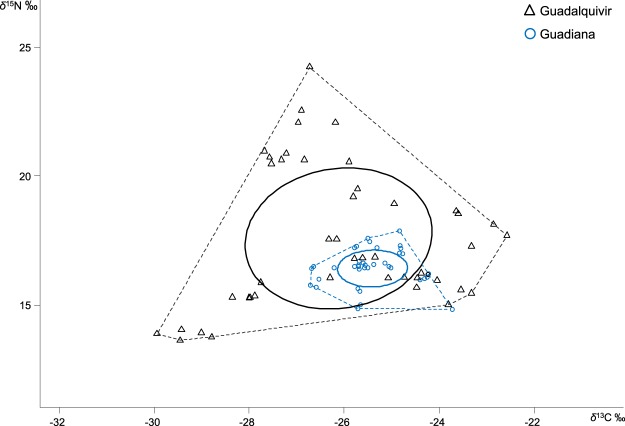
Trophic niche width according to the convex hull area (dotted lines) and standard ellipse areas corrected for a small sample size (SEAc) for the Guadalquivir (black lines) and Guadiana estuaries (blue lines). Triangles represent individuals of all the species measured in the Guadalquivir estuary, and circles represent species found in the Guadiana estuary.

Our results suggest that the higher human pressures found in the Guadalquivir estuary lead to more complex feeding pathways, as shown by a greater trophic niche width (−23.05 to −29.32 and 6.75 to 21.34 max and min values of δ^13^C and δ^15^N respectively in the Guadalquivir estuary while in the Guadiana they were −25.81 to −27.20 for δ^13^C and 5.83 to 17.28 for δ^15^N) and by the greater variability in organism position within isotope niche space (Figs 2 and 3). A greater distribution among species along the carbon axis suggests that primary consumers exploit organic matter of various origins, whereas the different nitrogen signals of secondary consumers suggest that they feed on different prey items (Fig. 2). There was greater intraspecific variability in isotope signals in this estuary, suggesting more variation in diet composition. In contrast, the similar isotope signals of secondary consumers in the Guadiana estuary suggest similarity in their diet composition, implying that they feed on the same organic matter sources.

**Figure 4:**
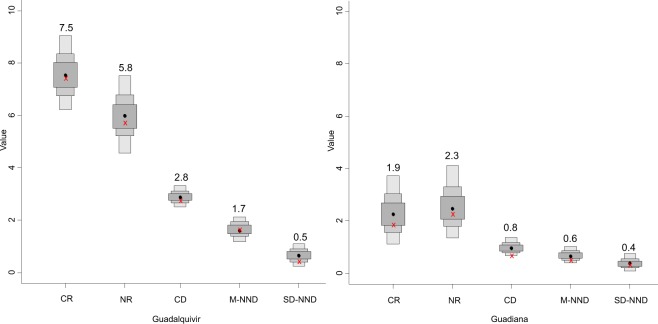
Bayesian results for the estuarine community-wide metrics that provide information on trophic diversity: carbon range (CR), nitrogen range (NR), mean distance to the centroid (CD) and trophic redundancy measured as the mean nearest neighbour distance (M-NND) and its standard deviation (SD-NND). Black dots are the modes, and boxes indicate the 50%, 75% and 95% credible intervals. The numbers above the red crosses represent the values of the crosses, which are the true population values.

The trophic structure of both estuarine communities, measured with the standard ellipse area (SEAc, where c indicates that the SEA was corrected for a small sample size) and the total area (TA), were distinct (Guadalquivir TA = 41.03 vs Guadiana TA = 5.74) (Fig. 3). The total overlap between the ellipses was the size of the Guadiana ellipse; it was much smaller and located inside the Guadalquivir SEAc (Guadiana SEAc = 1.66 vs Guadalquivir SEAc = 16.89, Fig. 3). Furthermore, the probability that the Bayesian standard ellipse (SEA_B_) value of Guadalquivir was larger than the SEA_B_ value of Guadiana was 100% (Supplementary Fig. S1). The community metrics^18^ also showed large differences between estuaries (Fig. 4). All indices were smaller in the Guadiana estuary than in the Guadalquivir estuary. Therefore, the smaller SEA and mean distance to the centroid (CD) in the Guadiana estuary suggest a more compact food web and lower trophic diversity than in the Guadalquivir^47^. Trophic redundancy (low mean nearest neighbour distance (M-NND)) and its standard deviation (SD-NND) were also higher in the Guadiana estuary.

Individuals within populations can exhibit variation in their trophic niche^20^. Variance in stable isotope values among individuals within populations can be used as a proxy of diet variation. This individual specialization is determined by biotic interactions such as predation and competition and by resource diversity^20,48^. Experimental and comparative studies suggested that while intraspecific competition increases individual specialization, interspecific competition reduces it^48^. In contrast, an increase in ecological opportunity, defined as the diversity of available resources, favours individual specialization^48^. Although other factors can influence the intraspecific variability of SIA, such as the size of individuals^49,50^, the higher diversity of resources in the Guadalquivir estuary seems to explain the higher intraspecific variability. Moreover, species that feed on more than one trophic level can also increase the intraspecific variability^49^, which is consistent with the higher trophic diversity found in the Guadalquivir estuary.

Organisms in the Guadalquivir food web tended to have higher δ^15^N values than those in the Guadiana food web, as reflected by the higher SEA position on the nitrogen axis of the Guadalquivir estuary. The high nitrogen range (NR) value in the Guadalquivir estuary suggests more trophic levels and more energy transfer to higher trophic levels^51^, and could be explained by the higher nutrient load. The similarity of the δ^15^N values of sedimentary organic matter (SOM) and vegetal matter in the two estuaries, in contrast to our hypothesis, suggests that this baseline variation does not drive the differences. However, the quick turnover rate of nitrogen in the primary producers could hide differences between estuaries in those resources since these values could be a snapshot of the temporal variation in the two systems^52^. Organisms at higher trophic levels integrate the stable isotope values of primary producers over time in their tissues, which helps to capture potential variation in basal carbon isotope signatures^52^. For this reason, the use of fish larvae and juveniles in planktonic communities is often a better long term indicator of water nitrogen content^27,53^.

Individual species, however, exhibited small differences between estuaries (Table 1). The variation was greatest in the large size class of the anchovy (*Engraulis encrasicolus)*, the mysid (*Neomysis integer*) and the shrimp (*Palaemon sp.)*. Differences in trophic position between estuaries of the mysid species *Neomysis integer* could be explained by the addition of an intermediate consumer to its diet or a change in its degree of trophic omnivory in the Guadalquivir estuary^54^. *N. integer* has been described as an opportunistic omnivore species that utilizes mesozooplankton and detritus as food sources and is able to feed on juveniles of the other mysid species such as *Mesopodopsis slabberi*^55^. In contrast, *M. slabberi*, which showed no difference between estuaries, feeds mostly on primary producers^56^. In the case of the anchovy *E. encrasicolus*, only the largest size class showed enriched δ^15^N in the Guadalquivir estuary. This result could be explained by the addition of an intermediate consumer such as the juveniles of the invasive isopod *Synidotea laticauda* which were only found in the Guadalquivir estuary (Fig. 2) or a change in trophic omnivory. In the Guadalquivir estuary, *E. encrasicolus* larvae change their diet from copepods to mysids as they grow^57^, which could explain the δ^15^N differences. Additionally, these differences could be explained in part by *E. encrasicolus* entering these two estuaries in early life history stages from the same spawning area^58^ and leaving in a later developmental period^57,59^. Consequently, the larger individuals of this species would feed longer on enriched sources in the Guadalquivir and would show larger differences with respect to the Guadiana than would the smaller individuals.

**Table 1:** δ^13^C and δ^15^N means (standard deviations) per species in the two estuaries and PERMANOVA pairwise results (t) of the comparison of each species between the two estuaries (* and ** indicate significant differences of species in their isotopic signal, where *p < 0.05 and **p < 0.01). Trophic position: producer (P), primary consumer (C1), and secondary consumer (C2).

*Species*	Organisms Type	Guadalquivir	Guadiana	t	Guadalquivir	Guadiana	t
		δ^13^C	δ^13^C		δ^15^N	δ^15^N	
***Engraulis encrasicolus*** **large**	C2	−26.33 (0.57)	−25.14 (0.84)	2.61*	19.5 (2.23)	16.64 (1.05)	2.6*
*Engraulis encrasicolus* medium	C2	−25.85 (0.4)	−25.78 (0.27)	2.75*	17.12 (0.4)	16.49 (0.04)	0.68
*Engraulis encrasicolus* small	C2	−24.54 (0.39)	−25.29 (0.23)	5.92**	16.07 (0.1)	16.92 (0.56)	8.52**
*Pomatoschistus sp*.	C2	−23.53 (0.92)	−24.82 (0.03)	3.13*	18.39 (0.48)	17.28 (0.36)	4.15**
***Palaemon sp*** *.*	C2/C1	−25.81 (1.45)	−24.29 (0.07)	2.34*	21.34 (2.62)	16.1 (0.1)	4.46**
*Synidotea laticauda*	C2/C1	−23.05 (1.72)			14.82 (1.4)		
***Neomysis integer***	C2/C1	−27.46 (0.19)	−25.62 (0.06)	20.62**	20.74 (0.2)	16.55 (0.12)	40.34**
*Mesopodopsis slabberi*	C1	−27.99 (0.22)	−25.69 (0.03)	22.84**	15.42 (0.26)	15.34 (0.37)	0.41
*Copepods*	C1	−29.32 (0.44)	−26.63 (0.07)	13.39**	13.85 (0.16)	16.07 (0.37)	12.26**
*Corbicula fluminea*	C1		−26.93 (0.76)			14.28 (0.39)	
Oligohaline vegetal matter	P	−24.89 (0.60)	−26.72 (0.02)	8.98**	10.25 (2.53)	12.22 (0.37)	1.92
SOM	P	−25.78 (0.05)	−27.2 (0.06)	28.96**	6.75 (0.52)	5.83 (0.06)	3.32

In the Guadiana estuary, the δ^13^C values of all organisms were similar, as indicated by the low carbon range (CR) and high redundancy values. These similar values could indicate the use of freshwater or brackish water particulate organic matter (POM), rather than marine inputs, as a carbon source; the latter would have more enriched carbon values. The similar isotope signatures between consumers suggest a more confined suite of prey resources than in the Guadalquivir estuary; these prey resources potentially include copepods and mysids (*Mesopodopsis slabberi*). Potential high trophic redundancy in this estuary showed by the Layman’s metrics could indicate a higher capability of species to play similar trophic roles and could support resistance to disturbances without the loss of connectivity in the food web^60^. However, pairing this information with stomach content analysis is necessary to truly assess trophic redundancy^60^. Moreover, the small number of links between primary consumers and secondary consumers may lead to an increase in fragility^61^. Niche width collapse and homogenization in the energy flow pathway have been described in fragmented systems, resulting in a less stable food web structure^15^. Low SEA values and low trophic diversity in estuarine fish food webs have been related to the low availability of aquatic producers as a consequence of the high level of suspended solids, which would limit primary production, which is not the case for Guadiana^47^. The simplification of the complex food web is also related to an increase in the vulnerability to environmental changes that can affect productivity and secondary extinctions^62^.

Conversely, in the Guadalquivir estuary, consumers showed greater differences in their carbon stable isotope signals, a greater niche width, more trophic diversity and lower redundancy values. These differences could indicate that the organic matter sources are different^63^. Thus, the Guadalquivir estuary would have a relatively more reticulated food web with multiple trophic pathways towards upper-level consumers.

Since the high turbidity present in the Guadalquivir estuary limits primary production, the allochthonous organic matter inputs in the oligohaline zone may be an important basal source. This detritus contribution can compensate limited phytoplankton production in highly turbid estuaries^64,65^. In the maximum turbidity zone of estuaries (MTZ), primary production was primarily bacterial, fed by detrital terrestrial and estuarine organic matter^64,66^. In the MTZ sediment, where organic matter aggregates by flocculation^65^, particles act as substrates for microorganisms that serve as prey for protozoa and other microorganisms^67^; the detrital energy is thus transferred to copepods and can also act as a food source for mysids^65^. The high abundance of copepods and mysids^64,66^ is supported by energy from detrital sources, which differs from nutritionally poor systems, in which food webs are based on algae in lower-turbidity areas^66^. This is also the case for the Guadalquivir estuary, in which the MTZ is located within the oligohaline zone^55^. In this estuary, the high biomass of copepods and mysids who feed on these detrital sources would support fish larvae and other crustaceans^68,69^. This finding agrees with those of other studies that have found that the turbidity maximum zone is a significant nursery area that positively influences fish growth and condition^66,70^. In addition, a positive relationship between the number of organic matter basal sources and fish production^71^ has been suggested.

These results could also explain the differences between estuaries in the mysid species; when detritus is present, *M. slabberi* shows a detritivorous/herbivorous tendency, and *N. integer* exhibits omnivorous behaviour with a carnivorous feeding tendency^64^. These authors described a 2- or 3-stage route from bacteria and vegetal matter to copepods. This route agrees with the high isotopic signatures of nitrogen of some species in the Guadalquivir estuary. In contrast, *M. slabberi* probably directly feed on detritus or phytoplankton, which would explain the lack of differences between estuaries in terms of the trophic enrichment factors.

The greater trophic niche width of the planktonic community, as well as the higher trophic diversity, could thus be explained by the different organic matter sources that are present in the Guadalquivir estuary^28^. Furthermore, another possible organic matter source would be linked to microphytobenthos in Guadalquivir mudflats. This particular organic matter source has been reported in other estuaries as one of the principal basal sources for the pelagic food web^72^. In contrast, the lower turbidity in the Guadiana estuary would permit higher phytoplankton primary production in the water column, which would be the base for copepods and *M. slabberi*, in turn sustaining all the secondary consumers. The smaller mudflats would also limit the contribution of microphytobenthos to the food web, but the overlapping values of carbon isotopes in the basal resources make it difficult to identify the main resources. Therefore, these conclusions are a first overview of organic matter origins for this two estuarine food webs.

The species found in each estuary were a good representation of the native planktonic macrofaunal communities. The same species were found in both estuaries except of the invasive isopod *S. laticauda* mentioned before (found in the Guadalquivir estuary) and the invasive clam *Corbicula fluminea* (found in the Guadiana estuary)^73–75^. Stable isotope studies assessing the effects of invasive species have been more frequently used in terrestrial and freshwater systems^76^. However, recent studies have applied stable isotopes to assess the effects of invasive species on marine ecosystems^23^. For example, the trophic niche of the benthic food web was wider in sites invaded by the macroalgae *Caulerpa cylindracea* than in non-invaded sites due to an increase in the diversity of basal resource pools^25^. In contrast, another study found a compacted food web structure in *Caulerpa prolifera* meadows^77^. There is a large degree of overlap in the utilization of basal sources, which is related to intra- and interspecific competition^77^ and is characteristic of degraded systems^15^. In this sense, our results showed that the invasive species *S. laticauda* may have an effect on the food web in the Guadalquivir estuary. First, this organism has isotopic signatures similar to those found in small anchovies (*E. encrasicolus*), suggesting that they are potential competitors. This potential risk caused by an invasive species has been also suggested^78^. Second, small individuals of this isopod could act as prey and could explain higher nitrogen signatures found in secondary consumers in the Guadalquivir. Additionally, this species showed the most enriched carbon signatures (Fig. 2), suggesting that it feeds on microphytobenthos and/or more marine basal sources than other species, which would explain the higher trophic diversity of the Guadalquivir estuary. In contrast, the impacts of *C. fluminea*, the invasive species found in the Guadiana estuary, do not appear to be readily visible in this study, and further investigation into the impacts of this species on planktonic food webs is certainly warranted.

Although our results show that the isotopic signal of nitrogen in the planktonic community generally seems to reflect the higher anthropogenic pressure present in the Guadalquivir estuary (Table 1), other factors could contribute to these differences. In contrast to our hypothesis and previous related research^16^, even though the Guadalquivir estuary has higher human pressure, the food web is more complex, with more feeding pathways, a greater niche width, more trophic diversity and lower trophic redundancy than the Guadiana estuary. The different organic matter sources present in the Guadalquivir estuary and the detrital processes in the MTZ could explain these distinctions. These results could be related to the higher nitrogen loads in the Guadalquivir estuary, which would have a positive effect on food web consumers by improving the nutritional quality and palatability of basal resources^79^. This finding also agrees with Warry *et al*.^12^ that found higher trophic diversity and less redundancy in fish food webs in estuaries with high nitrogen loads and suggested that the same pattern may be found in systems where the nitrogen loads are high and there is not a single dominant organic source.

## Conclusions

This study concludes that the Bayesian approach to the “Layman metrics^18^” is a useful tool with which to detect ecological differences in food webs between estuaries under different human pressures, as has been demonstrated in other studies^47^. Furthermore, stable isotope analysis revealed differences in the trophic interactions of species that were present in both estuaries, which is important information which complements traditional species surveys^24^ by providing key, additional ecological information about differences between these estuaries. Because this study is a comparison made during the summer season, the results obtained must be considered carefully, keeping in mind that the conclusions obtained are applicable to the oligohaline zone. Nevertheless, the important trophic differences observed between these two systems allow us to extract some conclusions and also to point out some characteristics that would be worth being further investigated. Thus, more extensive research on the spatial and temporal variability of the origin of basal resources, as well as the bottom-up interactions of estuarine food webs and their relationships with other environmental factors, is needed to better understand the food web dynamics in both systems. Understanding the trophic interactions present in estuaries with a strong human presence is crucially important for defining proper management and conservation strategies^80^. Furthermore, the management of factors that influence an estuary, such as freshwater discharges and urban and agricultural wastes, can regulate inputs of basal resources and modulate a phytoplankton or detrital dominated food web.

One important environmental implication of this research is that, even under altered conditions, the community of the Guadalquivir estuary seems to have reached a comparatively complex structure, which ensures a high productivity and some important ecosystem services such as the nursery function^59,81^. A word of caution should be included here for the future environmental management of this estuary since, any change, even with the objective to improve the environmental quality, should be done slowly and closely monitored. For instance, the high and permanent turbidity in this estuary is a present concern and its reduction is a commonly claimed objective^82,83^. However, the possibility that introduced sediment could be partly associated with the main sources of carbon for the community (allochthonous organic matter), makes it advisable to proceed with any potential restoration measure with caution, since any abrupt change in the present equilibrium would probably affect the nursery function and the fisheries production in the nearby marine areas. Assessing trophic structure and its relationships with other factors is crucial for understanding the consequences of increasing human pressure on estuaries.

## Methods

### Sampling

To eliminate any seasonal bias, sampling was carried out in summer 2017. Sampling was performed in the oligohaline zone of the estuaries to avoid marine influences on species isotopic niche breadth and assemblage architecture^12^. Several samples of the planktonic community were collected along the oligohaline zone to characterize the possible variation within this area. Samples were collected with a zooplankton net with a 1-m mouth diameter and 1000-µm mesh size. Twelve oblique tows were performed from the surface to the bottom during flood tide in the main channel at a constant speed of 2 knots. Parallel, copepods were collected with a bongo net with a 200-µm mesh size following the same process. Samples of soft bottom community were collected with a van Veen grab (0.05 m^2^) although only the clam *C. fluminea*, found in the Guadiana estuary, had enough biomass for isotopic characterization. All organisms were sorted by species, and juvenile anchovies (*Engraulis encrasicolus*) were sorted into three size classes: large, juveniles of 34.2 to 43 mm; medium, postlarvae of 27.8 to 31.5 mm; and small, postlarvae of 18.5 to 25.1 mm. The organisms were transferred to the laboratory in refrigerated containers and kept alive for 24 h to allow stomach evacuation to avoid any possible interference with the isotope signatures of their prey. Permission to gather the samples was obtained from the local authority “Consejeria de Medio Ambiente de Andalucía”. There are no ethical concerns associated with our study based on Directive 2010/63/UE and order ECC/566/2015. Primary producers were sampled by first sieving the sample collected with the bongo net through a sieving column and then selecting the vegetal matter under the stereoscopic microscope. Three sediment organic matter samples were collected with the van Veen grab from the uppermost 2 cm of the sediment. Other possible primary producers were extracted from the literature (see supplementary information).

### Isotope analyses

We rinsed animal and plant samples with distilled water. Muscle tissue samples of fish, clams and shrimp abdomens were used for isotopic analysis. Multiple organisms (>50) were pooled when the individuals had low biomass values (Supplementary Table S2). Samples were dried at 60 °C and ground to a powder. Sediment samples were acidified with 0.1 M HCl to remove carbonates and then oven dried. Organismal tissues were not acidified to avoid alterations in isotopic values^84^. Subsamples of powdered materials were weighed to the nearest 0.3 μg and placed into tin capsules for δ^13^C and δ^15^N analysis. Isotope analyses were carried out at the Laboratorio de Isótopos Estables of the Estación Biológica de Doñana (LIE-EBD, Spain; www.ebd.csic.es/lie/index.html). All samples were combusted at 1020 °C using a continuous flow isotope-ratio mass spectrometry system with a Flash HT Plus elemental analyser coupled to a Delta-V Advantage isotope ratio mass spectrometer via a CONFLO IV interface (Thermo Fisher Scientific, Bremen, Germany).

To investigate changes in trophic diversity within both estuaries, analysis of community niche space was performed using a novel Bayesian approach with the metrics proposed by Layman *et al*.^18^ for quantitative comparison of food webs^85^. This method returns a posterior distribution of estimates of the original metrics, which include the δ^13^C range (CR), δ^15^N range (NR), mean distance to the centroid (CD), mean nearest neighbour distance (M-NND) and SD of the M-NND (SD-NND). The Bayesian inference technique provides measures of uncertainty for these metrics reported as sampling error for the estimates of the means. Thus, the technique permits robust statistical comparisons to be made between communities independently of the number of groups within the communities^47,85^. Briefly, the CR is indicative of niche diversification at the base of food webs. The NR is a representation of the vertical structure of a food web, and larger ranges suggest more trophic levels and a greater degree of trophic diversity. The CD provides a measure of the average degree of trophic diversity within a food web. M-NND represents trophic redundancy, and food webs with species with similar trophic ecologies will show smaller values. Finally, SD-NND is a measure of the evenness of the food web, and large values suggest more diversification of trophic niches (see^18^ for more details).

The total convex hull area (TA) and the standard ellipse area (SEAc) were also calculated (c indicates that the SEA was corrected for a small sample size). The two metrics were estimated as quantitative proxies of the isotopic niche width, although the SEA is less sensitive to outliers and sample size than the TA^85^. Differences in the SEAc between the communities were evaluated via Bayesian interference (SEA_B_) according to^85^. All measures were calculated using the SIBER package in R.

Differences in the isotopic values between estuaries were investigated using two-way permutational multivariate analysis of variance (PERMANOVA)^86^. Each δ^13^C and δ^15^N isotope variable was analysed based on two fixed factors: estuaries (Guadalquivir (GDQ)/Guadiana (GDN)) and species. Posterior pairwise tests were used to test for differences between species in the estuaries. The Monte Carlo P-value was used instead when small unique values in the permutation distribution were available (<100)^87^. The tests were based on Euclidean distance matrices of the untransformed data using 9999 permutations. Statistical analyses were conducted using Primer v.6 and PERMANOVA (Primer-E Ltd., Plymouth, UK).

## Supplementary information

Supplementary INFO

## Data Availability

The datasets generated during and/or analysed during the current study are available from the corresponding author on reasonable request.
