# Supplementary material for: Human pressures on two estuaries of the Iberian Peninsula are reflected in food web structure
Source: Sci Rep. 2019 Aug 8;9:11495. doi: 10.1038/s41598-019-47793-2 (PMC6687818; doi:10.1038/s41598-019-47793-2)
Supplement: Supplementary file 1 — Supplementary INFO [file 41598_2019_47793_MOESM1_ESM.pdf]

Human pressures on two estuaries of the Iberian Peninsula are reflected in food web structure

I. Donázar-Aramendía*[1], J.E. Sánchez-Moyano[2], I. García-Asencio[2], J.M. Miró[1], C. Megina[3], J.C. García-Gómez[1]

SUPPLEMENTARY MATERIAL

Table S1. Carbon and nitrogen stable isotope signatures of primary producers in Guadalquivir and others close estuaries extracted from the literature. POM (Particulate organic matter), SOM (sediment organic matter).

Estuaries		Fresh water POM	Brackish water POM	Marine water POM	Microphytobenthos	SOM	Phytoplankton	References
Guadalquivir	$\delta^{13}C$		-26		-20.5	-24		1
	$\delta^{15}N$		6.37		9.5	10.9		
	$\delta^{13}C$	-26.5	-26	-20	-22			2
	$\delta^{15}N$	-0.5	2.5	9				
Tagus	$\delta^{13}C$		-22.7		-20.8	-24.1		3
	$\delta^{15}N$		10.8		14.3	16.2		
	$\delta^{13}C$	-24.8	-23.5	-21.3	-17.6	-20		4
	$\delta^{15}N$		3		6	6		
Minho	$\delta^{13}C$	-27.8	-27.4	-21.5	-24	-28	-38.5	5-6
	$\delta^{15}N$	4.9	5.3	5.2	7.3	0.2	5	
Lima	$\delta^{13}C$			-21.8				6
	$\delta^{15}N$			5.7				
Mira	$\delta^{13}C$		-23.3			-19.4		3
	$\delta^{15}N$		8.9			5.8		
Gironde	$\delta^{13}C$	-27.7	-26.7	-22.1	-23.9	-25.5	-34.5	7
	$\delta^{15}N$	5.8	6.3	9.7	9.1	5.3		
Charente	$\delta^{13}C$	-29.2	-25.3		-16.2			8
	$\delta^{15}N$							
Mondego	$\delta^{13}C$			-22.5	-14			9
	$\delta^{15}N$			6	6			

Table S2. Processing method, number of analysis and number of organism included in each analysis for each species.

Species	Processing method	Weigh (mg)	Analysis	Number per replies
Engraulis encrasicolus large	Individual	0.3	5	1
Engraulis encrasicolus medium	Individual	0.3	5	10
Engraulis encrasicolus small	Pool	0.3	5	> 10
Pomatoschistus sp.	Pool	0.3	5	5
Palaemon sp.	Pool	0.3	5	> 7
Synidotea laticauda	Pool	0.3	5	> 7
Neomysis integer	Pool	0.3	5	Pool
Mesopodopsis slabberi	Pool	0.3	5	Pool
Corbicula fluminea	Individual	0.3	5	1

Copepods	Pool	0.3	5	Pool
Oligohaline vegetal matter	Pool	1	5	Pool
SOM	Sediment	15	3	1

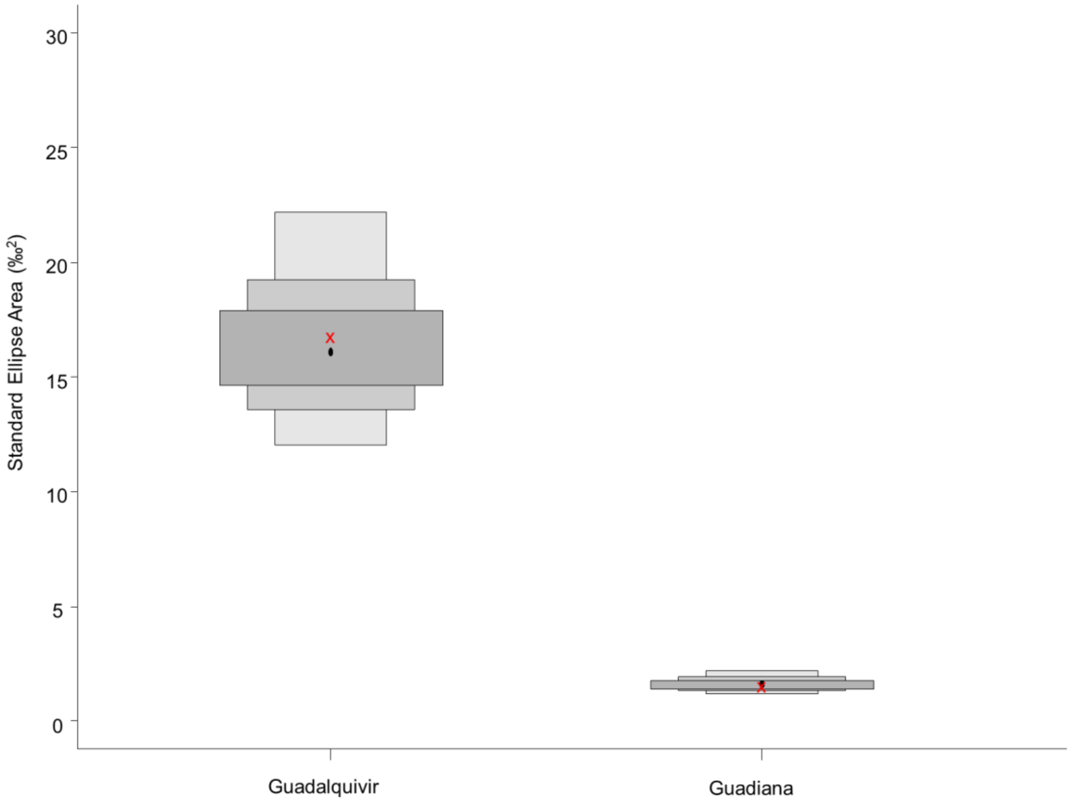

Figure S1. Density plots showing the credibility intervals of the Bayesian standard ellipse areas (SEA_B). Black circles are the SEA_B modes, and boxes indicate the 50%, 75% and 95% credible intervals. Red crosses are the true population values.

REFERENCES

1. González-Ortegón, E. & Drake, P. Effects of freshwater inputs on the lower trophic levels of a temperate estuary: Physical, physiological or trophic forcing? *Aquat. Sci.* **74,** 455–469 (2012).
2. Walton, M. E. M. *et al.* The effect of water management on extensive aquaculture food webs in the reconstructed wetlands of the Doñana Natural Park, Southern Spain. *Aquaculture* **448,** 451–463 (2015).
3. França, S. *et al.* Assessing food web dynamics and relative importance of organic matter sources for fish species in two Portuguese estuaries: A stable isotope approach. *Marine Environmental Research* **72,** 204–215 (2011).
4. Vinagre, C., Salgado, J., Costa, M. J. & Cabral, H. N. Nursery fidelity, food web interactions and primary sources of nutrition of the juveniles of Solea solea and S. senegalensis in the Tagus

estuary (Portugal): A stable isotope approach. *Estuarine, Coastal and Shelf Science* **76,** 255–264 (2008).

5. Dias, E., Morais, P., Antunes, C. & Hoffman, J. C. Linking terrestrial and benthic estuarine ecosystems: organic matter sources supporting the high secondary production of a non-indigenous bivalve. *Biological Invasions* **16,** 2163–2179 (2014).
6. Baeta, A. *et al.* Use of stable isotope ratios of fish larvae as indicators to assess diets and patterns of anthropogenic nitrogen pollution in estuarine ecosystems. *Ecological Indicators* **83,** 112–121 (2017).
7. Baeta, A. *et al.* Use of stable isotope ratios of fish larvae as indicators to assess diets and patterns of anthropogenic nitrogen pollution in estuarine ecosystems. *Ecological Indicators* **83,** 112–121 (2017).
8. Modéran, J., David, V., Bouvais, P., Richard, P. & Fichet, D. Organic matter exploitation in a highly turbid environment: Planktonic food web in the Charente estuary, France. *Estuarine, Coastal and Shelf Science* **98,** 126–137 (2012).
9. Rossi, F., Baeta, A. & Marques, J. C. Stable isotopes reveal habitat-related diet shifts in facultative deposit-feeders. *Journal of Sea Research* **95,** 172–179 (2015).
